# Uncovering the lack of awareness of sand mining impacts on riverbank erosion among Mekong Delta residents: insights from a comprehensive survey

**DOI:** 10.1038/s41598-023-43114-w

**Published:** 2023-09-24

**Authors:** Dung Duc Tran, Nguyen Duc Thien, Kai Wan Yuen, Rachel Yu San Lau, Jingyu Wang, Edward Park

**Affiliations:** 1 National Institute of Education (NIE), Earth Observatory of Singapore (EOS), Asian School of the Environment (ASE), Nanyang Technological University (NTU), Singapore, Singapore; 2 Centre of Water Management and Climate Change (WACC), Institute for Environment and Resources (IER), Vietnam National University, Ho Chi Minh City, Vietnam

**Keywords:** Environmental sciences, Environmental social sciences

## Abstract

Global sand demand due to infrastructure construction has intensified sand mining activities in many rivers, with current rates of sand extraction exceeding natural replenishment. This has created many environmental problems, particularly concerning riverbank stability, which adversely affects the livelihoods of people in the Vietnamese Mekong Delta (VMD). However, sand mining’s social impacts in the region remain inadequately understood. Here we assess locals’ perception of sand mining activities in the VMD and its impacts on riverbank erosion. Residents living along the Bassac River, a hotspot of sand mining, were interviewed. Our results showed that while sand mining is perceived as destructive to the environment, few were aware of its role in worsening riverbank erosion. Only residents directly affected by riverbank collapse were aware of the implications of sand mining and its negative effect on bank stability, as they seem to have actively sought clarification. Our findings highlight the need for greater awareness and understanding among the locals regarding sand mining’s impact on riverbank stability.

## Introduction

River sand mining is the extraction of sand (and gravel) from rivers^1^. As a key construction material, it is an essential mineral for fulfilling national development agendas^2^. Economically, the construction industry has significantly contributed to the GDP of Asian countries (i.e., 52% in China, 34.2% in India, 43.3% in Russia, 28% in Japan, 39.2% in Thailand), European countries (i.e., 37.1% in Germany, 22.1% in Italy)^3^ and African nations (34% in Botswana)^4^. However, this has had adverse effects on the environment, contributing to both water and land pollution in rivers and residential areas due to the leakage of large amounts of waste and heavy metals^1^, and river morphology changes^5^. Notably, riverbank erosion has escalated in recent years, leading to severe property damage and affecting the well-being and security of local communities^6^. Despite these issues, public awareness of the environmental impacts of sand mining remains limited, hindering the implementation of effective mitigation and protection measures. This lack of awareness contradicts two specific Sustainable Development Goals established by the United Nations^7,8^, namely sustainable management of water resources and quality (Goal 6), and creation of inclusive, safe, flexible and sustainable residence (Goal 11).

**Figure 1:**
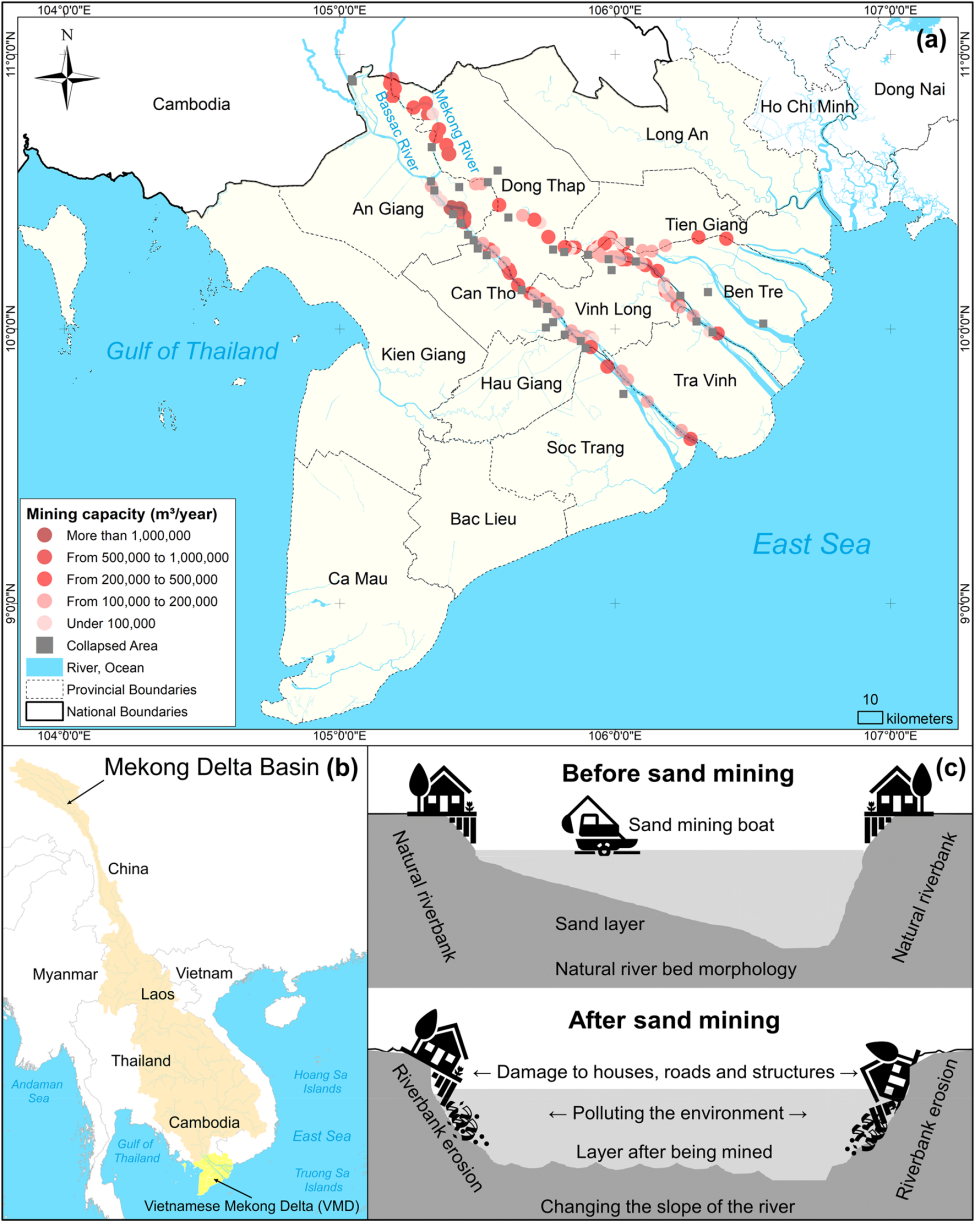
The problem of sand mining in the Vietnamese Mekong Delta (VMD). (**a**) Locations of riverbank erosion and sand mining capacity along the two main rivers of the VMD, with data source of sand mining capacity taken from provincial annual reports. (**b**) Geographical location of the VMD. (**c**) The physical mechanism of excessive riverbed sand mining on riverbank erosion. Maps are made using ArcGIS version 10.8 (https://enterprise.arcgis.com/en/).

In the Vietnamese Mekong Delta (VMD), the stability of the riverbank and the sustainability of people’s livelihoods are increasingly threatened by river sand mining activities. As a natural depositional site for sand and silt, this deltaic plain has been continuously mined since the 1980s to meet the high demand for construction sand today^9^. According to statistical data from the Ministry of Agriculture and Rural Development, as presented in their annual meeting with the World Wide Fund for Nature (WWF)^10^, excessive sand mining has led to 621 instances of riverbank collapse, with a total of 610 km of river banks along the Mekong River being eroded (Fig. 1). Among these collapses, 147 locations experienced “extremely severe” erosion (with a total eroded length of 127 km) while 137 sites suffered “severe” erosion (with a total eroded length of 193 km)^11^. These incidents have been attributed to prolonged river sand mining activities along the two main rivers of the VMD, the Mekong and the Bassac Rivers.

The Mekong and Bassac Rivers are hot spots of sand mining, primarily for local infrastructure development. According to reports, there are 82 companies licensed to extract 28 Mm^3^ of river sand annually along these two rivers, excluding the amount illegally extracted^11^. A recent study by Gruel et al.^12^ showed that an annual average of 42 Mm^3^ of sand (legal and illegal) was exploited from the two VMD rivers from 2015 to 2020. Based on previous calculations of sand entering the VMD and exiting to the sea, this amount of extraction has resulted in a sediment deficit amounting to 25 Mm^3^^11^. Such substantial losses contribute to lowered riverbeds, which increase flow velocity and sediment starvation, eventually worsening riverbank erosion. According to a report by the An Giang Department of Natural Resources and Environment in 2021, 181 km of the river bank was at risk of bank collapse, and the provincial government had to issue 56 warning sections to warn people of this threat^13^. In addition, more than 5,380 households that live along the river were in need of urgent relocation^13^. In Can Tho Province, though bank collapse was reportedly less severe, 226 sites of bank collapse were recorded between 2010 and 2020, with about 53 km deemed at risk of further bank collapse^14^. Despite increasing concerns of bank collapse associated with sand mining activities, provincial managers and policy makers have yet to respond effectively to address this issue.

To date, there have been several studies globally that have investigated people’s perceptions towards sand mining and their level of awareness towards sand mining-induced bank collapse^15–19^. Ratang^15^ explored miners’ perception of sand and stone mining activities in Nolokla village in Indonesia, with field observation methods and interviews of 21 miners. Results showed that miners valued the economic benefits of the sand and stone mining activities, despite acknowledging their negative impacts on land degradation, river water flow reduction and pollution. In another study in Trempealeau county, Wisconsin in the United States, Hammond^16^ carried out 269 online surveys with locals and 22 in-depth interviews with local community leaders, business owners, and landowners to understand public’s opinion. His results showed that 60% of the respondents were aware of the risks to their environment, health and socio-economic costs of frac sand mining development. Similarly, Sari et al.^17^ interviewed 30 people along the Mujur and Regoyo rivers of Indonesia to assess people’s perception on sand mining impacts. The study revealed a high level of public awareness regarding the positive (i.e., job, business, income) and negative impacts of sand mining (i.e., air pollution, erosion, river pollution, road damage). Most recently, Tri et al.^18^ interviewed 218 households in Dong Thap and An Giang Provinces of the VMD to quantify the social vulnerability of local communities affected by riverbank erosion along the Mekong and Bassac Rivers. Although they did not focus on assessing households’ perception, their findings revealed that 70% of the households surveyed were susceptible to riverbank erosion, while only a small fraction possessed the capacity to effectively manage the immediate consequences. Overall, studies on assessing people’s perceptions of riverbank erosion driven by sand mining were less common compared to those that focused on the environmental impacts of sand mining. In addition, there is a clear deficiency in understanding the social impacts of sand mining in Vietnam. For instance, while landslide or riverbank erosion in the VMD have been attributed to sand mining activities, the perception of locals towards sand mining remains largely unknown^20,21^. Although media reports have previously highlighted the insecurities of local residents affected by bank erosion and collapse, the association with sand mining activities along the river was not explicitly drawn.

Therefore, our study aims to assess the level of awareness among local residents regarding bank erosion or collapse, and their perception of ongoing sand mining activities in their vicinity. The Long Xuyen City in An Giang Province and Can Tho City in Can Tho Province were chosen as field sites as these regions have been documented hotspots of sand mining in the VMD. These locations serve as comprehensive study sites for understanding the social impacts of sand mining in the delta. In this study, we conducted a systematic literature review, field observations and in-depth interviews with 34 residents living along the Bassac River, where bank collapses previously occurred. We hypothesized that residents who are affected by riverbank erosion would be most concerned about riverbed sand mining and would recognise the importance of effective solutions for ensuring future sustainability. Our findings were then compared with relevant case studies from our systematic literature review. Overall, we intend for our study to provide local governments, managers and decision makers with a more holistic understanding of the implications of sand mining, and consequently implement more effective sand mining management strategies that integrate community insights. Arguably, while sand mining is important for national development, it is equally crucial to consider the health and wellbeing of local communities living in areas directly affected by sand mining.

## Methods

### Study area

**Figure 2:**
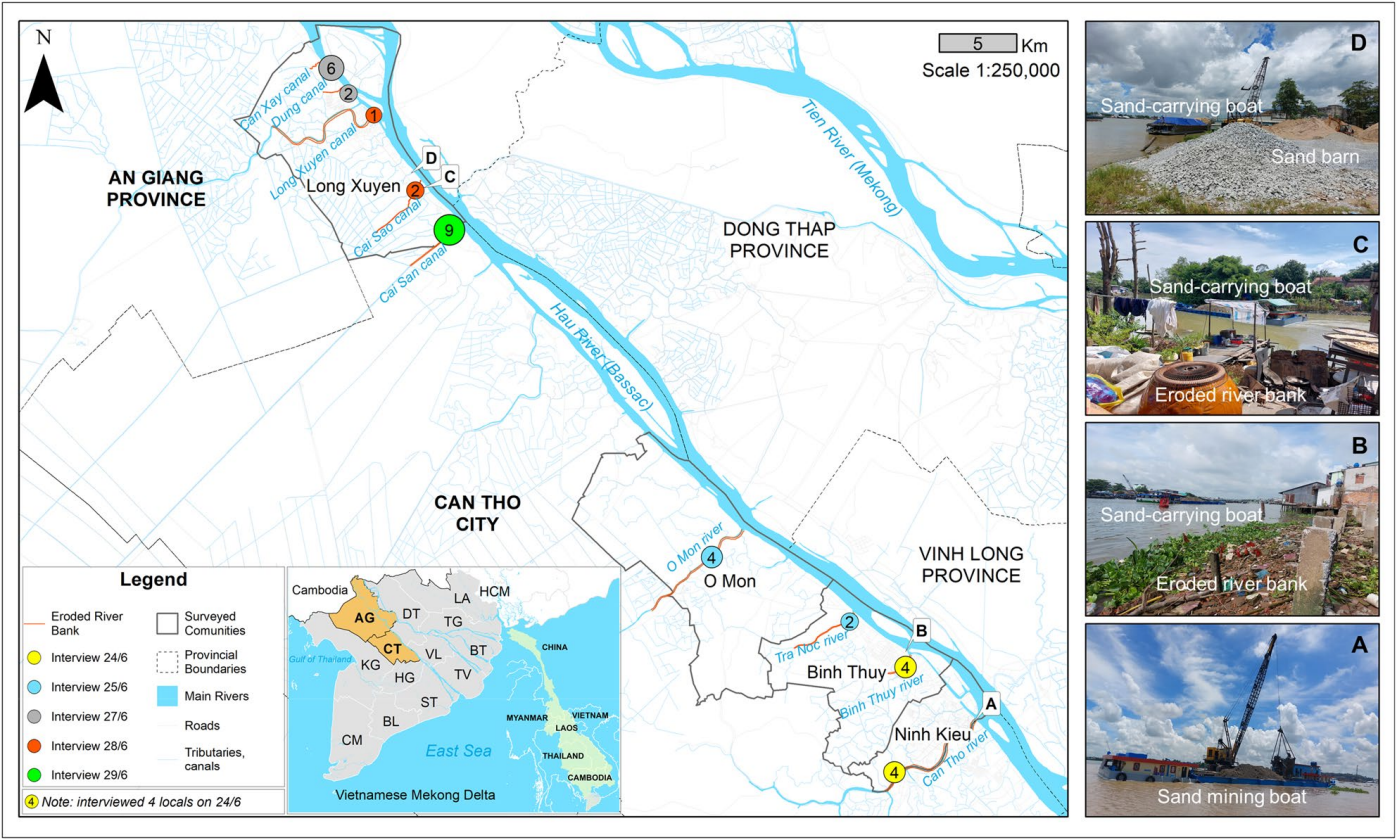
Four surveyed areas in Can Tho City (Ninh Kieu, Binh Thuy, O Mon) and An Giang Province (Long Xuyen). Map is generated using ArcGIS version 10.8.

An Giang Province and Can Tho City are located along the Bassac River where sand mining has been prevalent due to high demand for sand needed for national development projects and international export (Fig. 2). Along the length of the river in the two provinces, riverbank erosion has been increasingly reported as a result of morphological changes driven by (i) the hydropower dam developments upstream that reduce sediment supplies to the Vietnamese Mekong Delta (VMD), and (ii) the overexploitation of sand and gravel along the main rivers itself. Specifically, Binh et al.^22^ concluded that upstream dam development along the Mekong River has reduced sediment supply to the VMD by 74.1%. While the lack of sediment from upstream sources was concluded as the major cause of riverbank erosion, sand mining activities still contribute 14.8% to riverbank erosion annually. As a result, the riverbed gets hollowed while the river flow increases, eventually eroding the riverbank to rebalance the dynamics of the river.

We conducted our surveys across three districts of Can Tho City, namely Ninh Kieu, Binh Thuy, and O Mon. In addition, we also carried out field surveys in Long Xuyen City of An Giang Province. Our study sites are sand mining hot spot areas and riverbank erosion has been observed widely. Along the length of the Bassac River, over 1,000,000 m^3^ of sand can be legally extracted^23,24^. While anecdotal evidence suggests that riverbank erosion has worsened in recent years due to sand mining activities, it appears that most locals are unaware of the processes leading to bank erosion or collapse (Table 1).

**Table 1:** Characteristics of the study areas.

Variables	Can Tho City	Long Xuyen City
Area and population*		
Area (km^2^)	1440.0	115.3
Population (thousand people)	1247.0	286.1
Density (person/km^2^)	866	2485

Variables	Can Tho Province	An Giang Province
Labour*		
Number of employees	584,300	922,200
National economic contribution*		
GDP (billion VND)	103,225	74,297
Economic growth (%)	7.5	6.5
GRDP/person (million VND)	80.5	34.3
GDP (million $US)	4154.6	2990.3
GRDP/person ($US)	3240.0	1380.5
Exchange rate (2022)	*1US$* = *24845.5 VND*	
Sand mining**		
Permitted area for sand mining in 2013 (ha)	344	354
Permitted area for sand mining in 2020 (ha)	634	412
Allowable mining capacity in 2020 (Mm^3^)	2.52	5.26
Allowed volume of extraction in 2020 (Mm^3^)	7.56	11.95

*Statistical data are based on the 2018 statistical yearbooks of Vietnam.

**Statistical data are based on legal documents in 2013 and 2020 from the local government of Vietnam*.*

Table 1 presents the key characteristics of the study areas in Can Tho and An Giang, including their areas, population densities, number of employees, GDPs, growth rates, sand mining areas and the allowable rates of sand extraction. In terms of area, Can Tho City is much larger than Long Xuyen City, even though An Giang Province is nearly double the size of Can Tho Province. The population in Can Tho City (1247.0 thousand) is four times that in Long Xuyen (286.1 thousand); therefore, Long Xuyen has a higher population density than Can Tho. For national economic contribution, the economic growth and GDP of Can Tho Province is higher than An Giang Province. Regarding sand mining activities, both cities have seen an increase in permitted area for sand mining in 2020 (634 and 412 ha) compared to 2013 (344 and 354 ha). However, the allowable mining capacity of An Giang (5.26 Mm^3^) was higher than that in Can Tho (2.52 Mm^3^), due to the strict regulation of river sand exploitation by local governments following the issuance of National Resolution No. 23/2020/ND-CP in 2020.

### Research design and data collection

All methods were carried out in accordance with the relevant guideline of Nanyang Technological University (NTU) and approved by the NTU-Institutional Review Board (reference number IRB-2022-869). Standard informed consent was obtained from all residents/respondents participating in our study. Specific guidelines and details can be found in the NTU website https://www.ntu.edu.sg/research/research-integrity-office/institutional-review-board/guidelines.

A questionnaire was designed based on literature reviews and expert discussions. The questionnaire included five main sections, namely (i) personal information (name, place of residence, gender, age, occupation), (ii) role of river (role, purpose of use and recent changes), (iii) observation of sand mining activities (time and frequency of mining boats, transport boats and transport trucks) and (iv) people’s perception of sand mining (good or bad, what impact is involved, experience, solution and whether or not it is considered illegal). Respondents were primarily asked to provide their responses to a series of multiple-choice questions, which included various aspects of their personal experiences and opinions towards sand mining. For certain questions, our respondents were also asked to rank their responses from 1 to 5 (1 = not bad, 2 = not so bad, 3 = bad, 4 = pretty bad, 5 = very bad).

Interviews were conducted in June 2022. We randomly selected 34 residents who lived along the river, in close proximity to the annual riverbank erosion areas of Can Tho City (Ninh Kieu, Binh Thuy, O Mon) and An Giang Province (Long Xuyen). To ensure the reliability and comprehensiveness of the data collected, we targeted household heads or individuals who have been residing in the area for at least 10 years and possessed a good understanding of the local environment and way of life. By adhering to these criteria, we aim to gather substantial and meaningful information for subsequent data analysis. Each interview session lasted approximately 30 min. Rather than setting a specific number of interviewees for different collapsed areas, we continued the interviews until we reached the point of information saturation^25^, ensuring comprehensive data collection. For data analysis, descriptive statistics were carried out using Microsoft Excel and maps were created in ArcGIS version 10.8.

In addition, we did a systematic literature review on Google Scholar and Web of Science to obtain relevant case studies on people’s perceptions towards sand mining, as well as articles published from 2011 to 2022 that were related to sand mining and erosion. The following three key words were used: “sand mining”, “river erosion”, and “perception and awareness”. Of those three key words, “sand mining” was used instead of “river sand mining” as the latter yielded very few results. All relevant articles found in our literature search were given an initial screening before relevant studies were selected to be read in detail. Based on our literature search, we then compared our research findings with published information. A world map was also prepared in ArcGIS to show people’s perceptions towards sand mining, with the amount of sand exported by each country retrieved from https://oec.world/en/.

## Results

### Residents’ socio‑demographic characteristics

**Figure 3:**
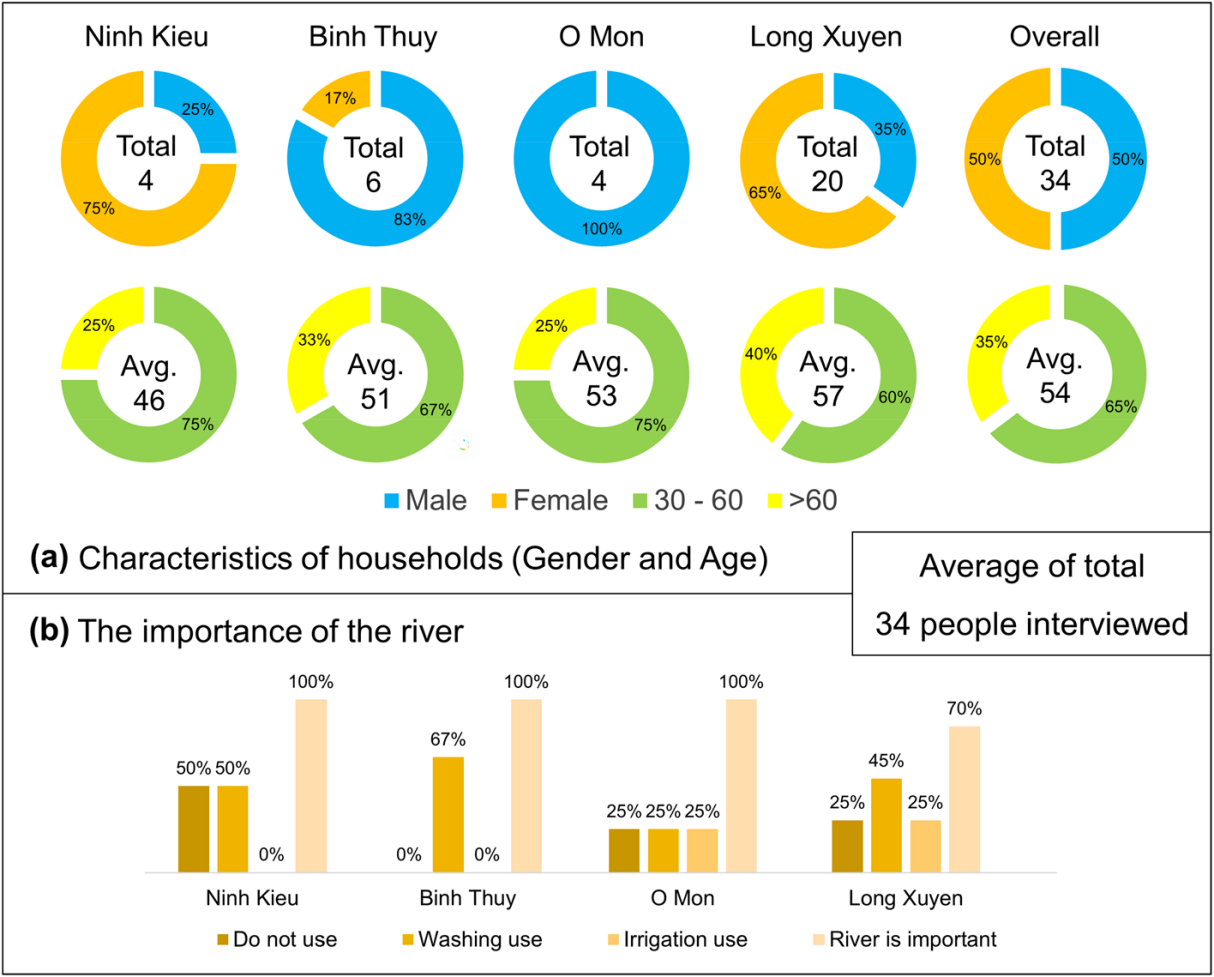
Basic information of residents interviewed in the study areas. (**a**) Characteristics of residents. (**b**) Their perception on the importance of the river.

The average age of the residents we interviewed in Can Tho and Long Xuyen Cities was 54. In Long Xuyen, 40% of the interviewees were over 60, though the gender ratio was 50% male and 50% female (Fig. 3).

Most respondents felt that the river played a vital role in their lives. All the residents we interviewed in Can Tho believed that the river brings many benefits to their daily activities, while 70% of An Giang residents felt that way. Most people use the river for washing purposes, with up to 67% reported in Binh Thuy. Unfortunately, the water quality in the VMD is affected by the excessive use of agrochemicals and salinity intrusion brought about by climate change induced sea level rise^26^.

### Observations on environmental issues caused by sand mining activities

**Figure 4:**
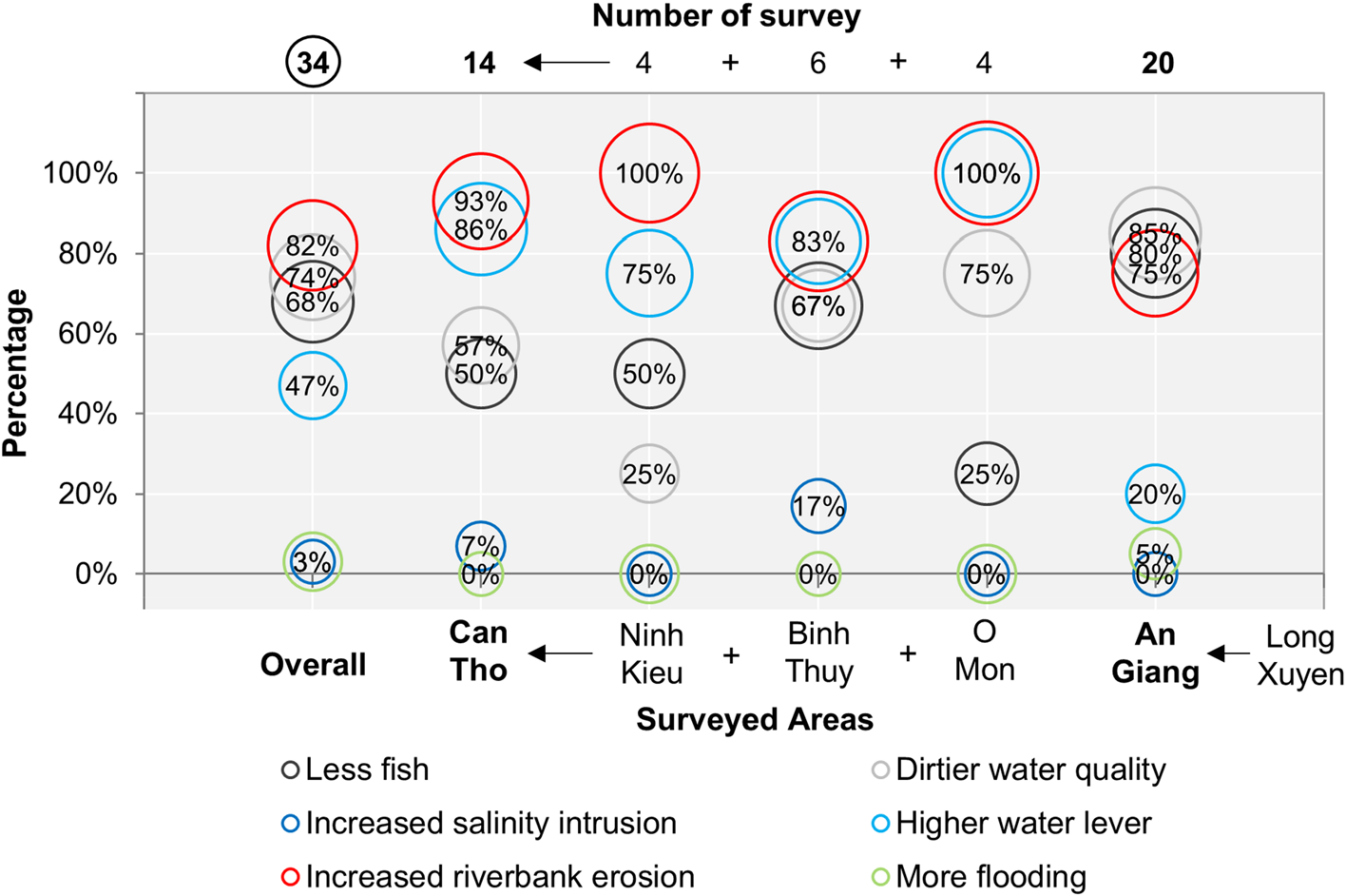
Environmental changes perceived by residents in An Giang Province and Can Tho City.

The problem of riverbank erosion was perceived as a common occurrence in Can Tho and An Giang Provinces, and respondents highlighted its increased frequency and scale in recent years. The most observed environmental issue associated with sand mining was “increased riverbank erosion” (with the highest average of all factors being 82%), followed by “dirtier water quality” (74%) and “higher water level” (68%). Most of the interviewed residents were affected and notably concerned about riverbank erosion, with 93% of our respondents in Can Tho and 75% in An Giang expressing concern over it (Fig. 4). These findings were consistent with the recent data from the Statistics Office of Can Tho^27^, which reported 9 incidences of riverbank erosion and a total of 268 m of eroded river bank. In An Giang^28^, there have been 28 cases of riverbank erosion with the total length of 1455 m.

**Figure 5:**
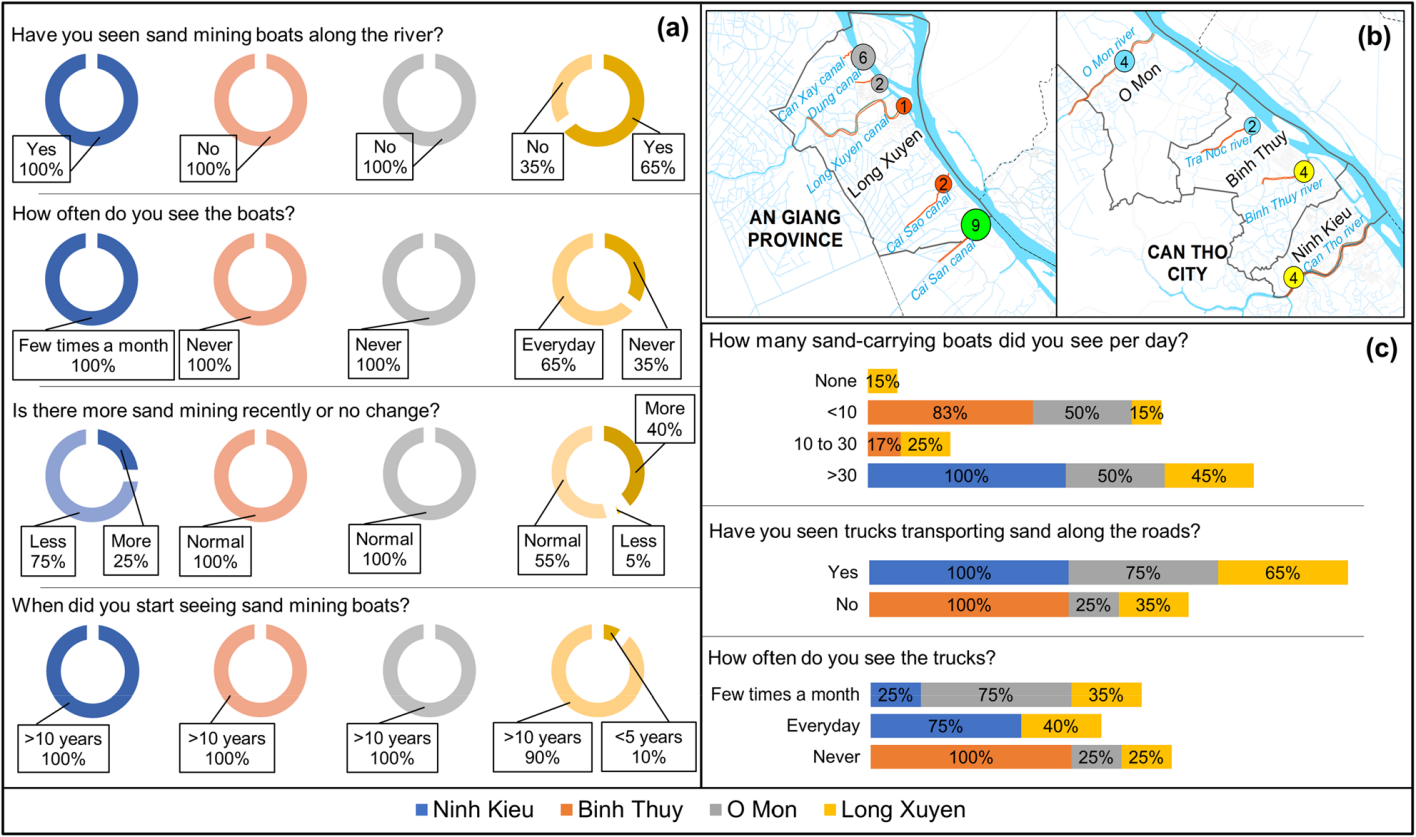
Awareness of residents based on their observations of sand mining activities. (**a**) Residents’ perception of observing sand mining boats. (**b**) Location of interview surveys. (**c**) Residents’ perception of observing boats and trucks transporting sand.

The assessment results of people’s observations on the time and frequency of mining boats, transport boats, and transport trucks showed that people were aware of sand mining activities in the area (Fig. 5). Specifically, 100% of interviewed respondents in Can Tho and 90% of those we interviewed in An Giang were aware that sand mining has existed for more than 10 years (the remaining % being newcomers). However, people’s awareness of sand mining activities was often dependent on the mining location and where they lived, as we found that 100% of residents interviewed in Ninh Kieu (4 interviewees in total here) and 65% in Long Xuyen had observed sand mining boats on the river crossing their living area. These two places belong to the VMD’s extensive waterway network, which explains the higher visibility of sand mining activities.

Most residents did not pay much attention to the frequency of sand mining activities through the years. Only 25% of interviewees in Ninh Kieu (1 over 4 interviewees in total) and 40% in Long Xuyen thought that sand mining happened more often in the area. In contrast, 75% of interviewed people in Ninh Kieu (3 over 4 interviewees) thought that the frequency of sand mining had decreased since sand mining activities have been banned by the provincial government of Can Tho. Meanwhile, for the remaining three surveyed areas, where sand mining was permitted, the people observed an increased frequency of sand mining activities. According to the 2020 statistics of the An Giang Department of Natural Resources and Environment, a total of 4,460,000 m^3^ of sand was allowed to be mined annually on the Bassac River^23^. Allowable mining sites were located along the river bounded by My Hoa Hung commune in Long Xuyen City and Cho Moi district. In Can Tho, about 1,370,000 m^3^ of sand was allowed to be mined annually along the sections of the river bounded by Thot Not, O Mon and Binh Thuy districts^24^.

As shown in Fig. 5, the majority of interviewees (averaging 72.5%, ranging from 45 to 100%) often saw more than 30 sand carrying boats along the river per day. The remaining few respondents in Long Xuyen (15%) chose “none” because they lived in an area that had experienced severe riverbank collapse and big boats were no longer permitted to travel on that section of the river. Similarly, when asked about the presence of sand-carrying trucks, some respondents also chose “no” because they lived in regions so severely eroded that heavy vehicles were restricted from using nearby roads. In addition, residents noted more sand carrying boats and trucks than sand mining boats. This could be due to the recent commencement of sand mining activities on the river nearby, and the routes taken by sand boats and trucks happened to be a distance away from where our respondents lived.

### Environmental consequences of river sand mining activities

**Figure 6:**
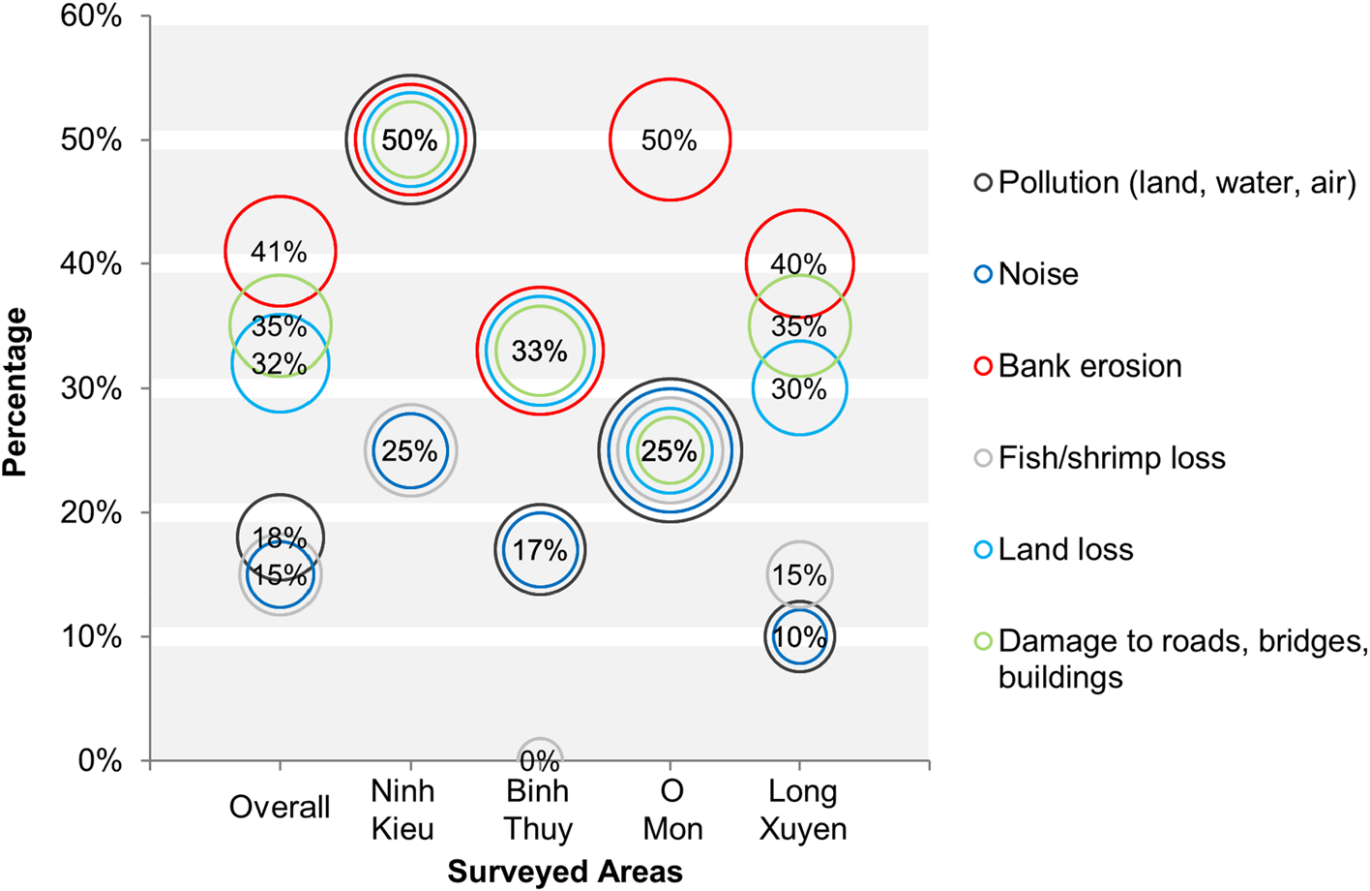
Residents’ perception on environmental issues caused by sand mining activities.

Residents were asked if they were aware of the impacts of river sand mining activities in terms of land, water, and air pollution, noise, bank erosion, fish/shrimp loss, land loss, and infrastructure damage (Fig. 6). Overall, a large number of residents (41%) believed that riverbank erosion was the consequence of sand mining activities, while 35% and 32% associated damage to roads, bridges, buildings and land loss to sand mining. Only a minority of residents mentioned pollution (18%), noise (15%), and fish/shrimp loss (15%) as environmental consequences of sand mining.

### Perception on sand mining in terms of its legalism, benefits and environmental management

**Figure 7:**
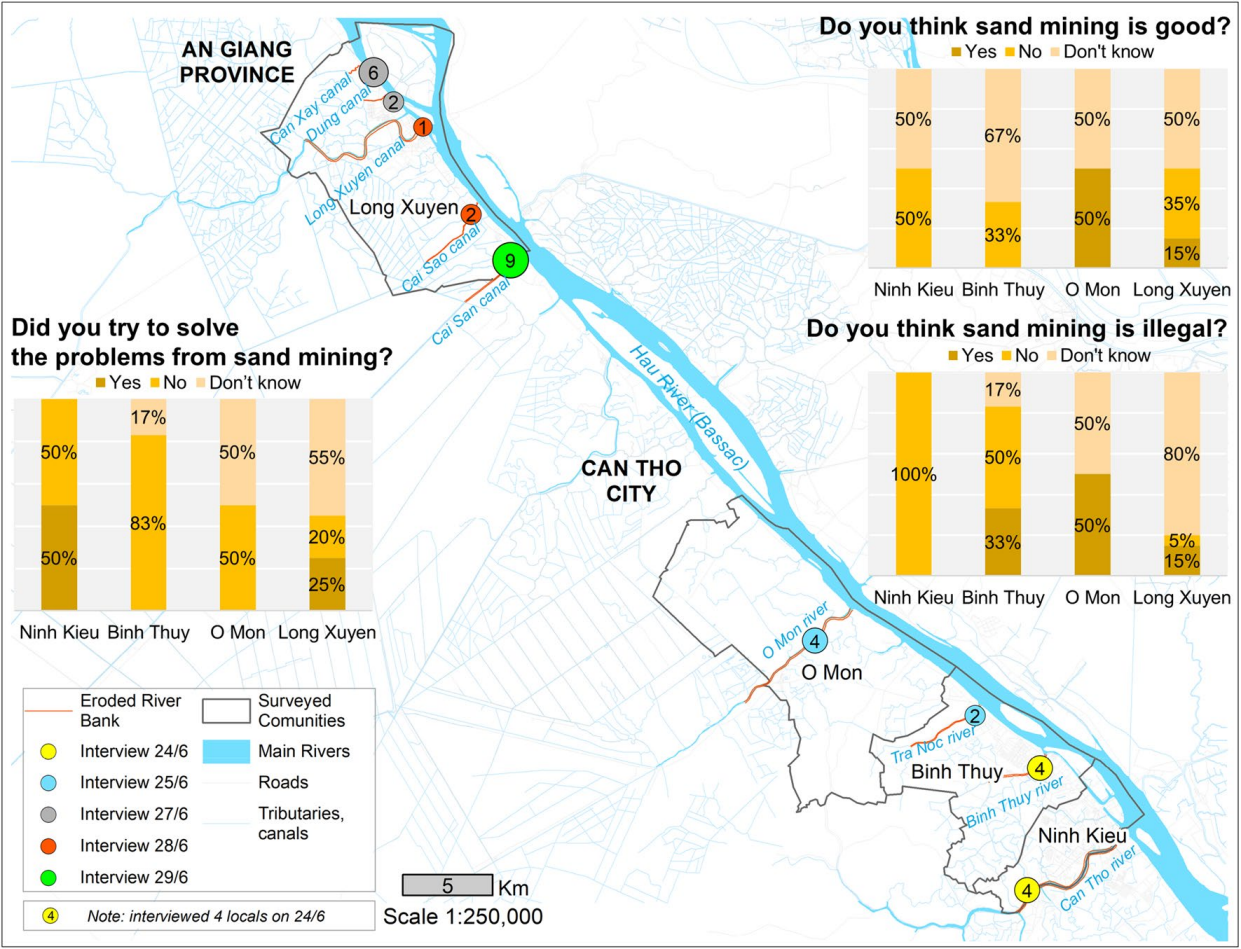
Residents’ perception on legalism, benefits and environmental management solutions of sand mining.

Residents of Ninh Kieu expressed mixed feelings about the impacts of sand mining. Among our respondents, 50% felt that sand mining was bad while the remaining 50% considered it beneficial as they perceived themselves capable of dealing with the environmental problems associated with sand mining (Fig. 7). When asked if sand mining in the area was illegal, 100% of people in Ninh Kieu (4 interviewees in total) selected the “no” option, as they believed that sand mining (licensed) is not prohibited in their area. In contrast, residents in the other areas were generally more aware of environmental issues and illegal mining activities.

More than 50% of residents in our four study areas had mixed views about sand mining as they felt that sand mining results in both beneficial and harmful impacts. They were aware that sand mining created more jobs and provided local communities with a higher income. Regarding the adequacy of addressing the problems of sand mining, more than 50% of respondents in the four surveyed areas indicated that they had accepted the problem without attempting to address them.

### Worldwide awareness on sand mining

**Figure 8:**
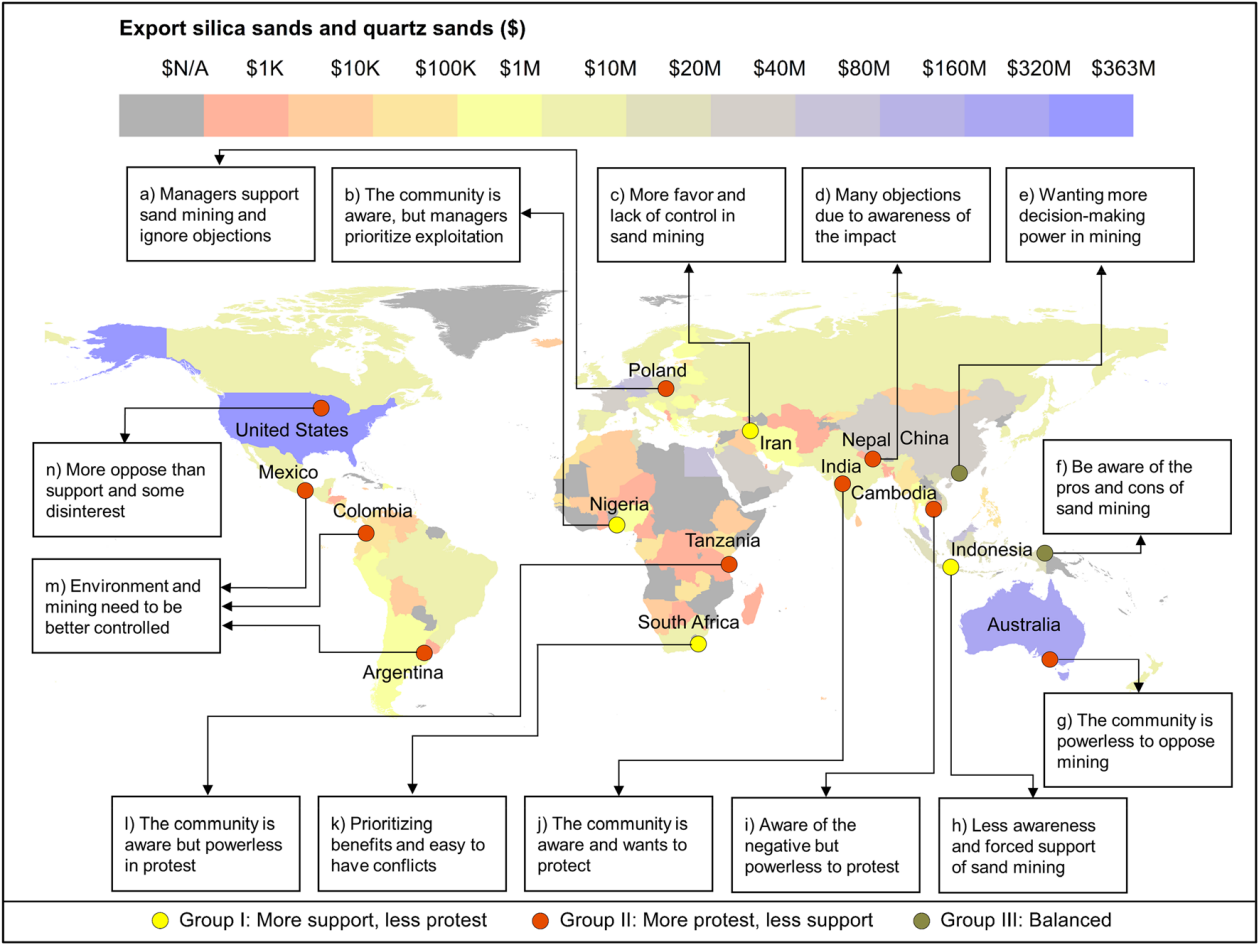
Map showing worldwide countries’ economic value ($ US) by sand export and perception of sand mining from articles. The United State and Australia are the two top sand export countries while Vietnam’s sand export is moderate. In boxes, perception of worldwide communities on sand mining by literature reviews was summarised based on publications including (**a**)^34^, (**b**)^35^, (**c**)^2^, (**d**)^30^, (**e**)^31^, (**f**)^15^, (**g**)^36^, (**h**)^37^, (**i**)^38^, (**j**)^39^, (**k**)^29^, (**l**)^40^, (**m**)^41^, (**n**)^16^. Map made by the authors using ArcGIS version 10.8. The amount of sand exported by each country was retrieved from https://oec.world/en/.

Based on our systematic literature review, we found 17 related papers which assessed the perception and awareness of local communities towards sand mining activities. These case studies were from 16 countries published from 2011 to 2022 (see Fig. 8 and more detail in Supplementary Table S1). Four out of 17 studies (26%, thereafter called Group I) found that local residents were supportive of sand mining activities. Meanwhile, eight out of 17 studies (47%, Group II) showed that their respondents were against sand mining activities. Lastly, only two case studies (11%, Group III) reported that half of their respondents were supportive, while the other half of their respondents were against sand mining. Although the scope of the remaining 3 studies (16%, Group IV intentionally not presented in Fig. 8) did not directly assess people’s perceptions of sand mining, they provided important physical and vulnerability assessments of sand mining activities in the VMD and beyond.

All 17 studies used interviews with local people to ascertain people’s perceptions of sand mining. Group I studies were supportive of sand mining and showed that sand mining had economic benefits. For instance, the study in South Africa^29^ reported that most respondents felt that sand mining brought jobs and income. Although sand mining has resulted in conflict, coastal erosion and health problems, these were deemed to be less important than being able to make a living. Another study in Iran^2^ showed that people could attain a good income from sand mining, although it was acknowledged that mining also resulted in environmental pollution, riverbank erosion, poverty increase, and a reduction in agricultural land area. As such, most respondents supported this activity, while only a minority of residents were concerned about the negative environmental externalities.

On the contrary, studies in Group II showed that environmental issues were perceived as a severe problem that outweighed increased income. For example, the study in Nepal^30^ found that illegal sand mining resulted in landslides and erosion, to which the affected areas can no longer be used. Other issues pertained to a lack of worker safety, reduced runoff, depletion of groundwater resources, and problems associated with drastically reduced flows and mudslides into farmland. Hence, 75% of local people opposed sand mining. The remaining 25% were supportive only because they worked in sand mining or related industries.

For the Group III^15,31^ case studies, respondents recognised the good and bad effects of sand mining activities.

Lastly, the studies in Group IV include assessments of the physical consequences of sand mining through adopting hydraulic modelling^32^ or assessing the livelihood vulnerabilities of people living in the VMD^18,33^.

## Discussion

### Reflections on people’s perception of sand mining-induced riverbank erosion

In the Vietnamese Mekong Delta (VMD), landslide and riverbank erosion were a common occurrence along the Mekong and Bassac River, and were often attributed to widespread sand mining activities in the region. However, few studies have provided an in-depth understanding of local perception of sand mining and its impacts. As such, we conducted thorough interviews with 34 residents along Bassac River to gauge their perception and awareness of sand mining in the VMD. We categorized respondents into three groups: (i) those significantly affected by landslides and erosion, (ii) those living near affected areas with uncertain awareness, and (iii) those with properties protected by concrete embankments.

Residents directly affected by landslides and riverbank erosion were generally aware of the risks and problems created by sand mining. Some respondents actively seek to understand the causes of bank collapse. By watching the news and reading newspapers or books, these respondents felt that sand mining was a major reason behind bank collapse, which also highlights the importance of media sources to increase local knowledge^42^. With the destabilisation of riverbanks, these people had to make the difficult decision to relocate to a more stable location or remain at risk of experiencing another bank collapse. Although the local governments had provided financial and technical assistance to support relocation, most respondents did not relocate due to the high cost and effort. Similar to a US Midwest flood retreat study^43^, affected individuals often avoid relocation due to high costs and effort, despite government financial assistance. Furthermore, some respondents also felt that the compensation by the government was insufficient and that the new location provided was inconvenient, similar to the findings by Huang et al.^44^. Notably, Krishnamurthy^45^ argued that forced migration threatens people’s physical safety and livelihoods, and this may explain why some respondents were reluctant to move even in the face of high risk.

As for respondents who lived near the landslide area but were not directly affected, they seem to be unclear about the causes and mechanisms associated with sand mining. Many assumed that the main cause of bank erosion/collapse was due to natural processes or strong waves generated by passing boats. Compared to those who were directly impacted by bank erosion and collapse, this group of people did not appear to be concerned about the causes of landslides or erosion. Consequently, these people were generally more supportive of the local sand mining industry. This could be detrimental in the long run as the lack of awareness can lead to inaction, leading to worse outcomes^46,47^.

In contrast to the above two categories, residents living in the areas protected by concrete embankments seem to be clearly unconcerned about the bank erosion/collapse, often continuing to build houses and living near the banks. However, such a false sense of security is dangerous as any future collapse entails greater losses^48^. This is highly probable in Vietnam, where many embankment projects are not synchronous due to limited budget and corruption^49^. Compared to synchronized embankment systems that are effective and stable^50^, unsynchronized embankment is only able to prevent landslides or erosion in one place while leaving other spots vulnerable to damage. Repairing the damage caused by a collapsed river bank will also be costly. Interestingly, this observation was highlighted by one respondent, who mentioned that embankment erosion can cause severe erosion in poorly maintained spots due to excessive loads on the surface.

Riverbank erosion, damage to infrastructure and land loss were the three commonly cited impacts of sand mining. Respondents who lived in a big city such as those in Ninh Kieu district of Can Tho City, were the most cognisant of this. However, we find that those who are not directly impacted by these negative impacts are often not bothered by sand mining activities. Our findings revealed that most people were aware of the negative impacts of sand mining on riverbank erosion but very few respondents were able to articulate how sand mining leads to bank erosion/collapse. Only 6 out of 34 respondents (18%) understood the physical mechanism by which sand mining contributes to riverbank erosion. These respondents lived in the locations where the riverbank had collapsed previously. They mentioned that sand mining on the main river changes the river’s morphology locally and even though they lived a distance away from any sand mining activities, the tele-connected nature of the river systems meant that they ended up being affected by the removal of riverbed sand^51^.

Our findings also showed that female residents were more observant of changes in the physical environment than males. Nevertheless, males were more aware of the linkage between sand mining and riverbank erosion in the area. Our results showed that 65% of female residents were aware of sand mining boats operating on the river (35% male) and 64% said that sand mining was not good (36% male). However, 83% of female residents stated that the river was not important to their lives (17% male). Out of the total of 34 interviews conducted, the six interviewees, who had some understanding of the physical mechanism of sand mining on riverbank erosion were all males. This may be explained by the limited mobility of the female residents who were either food sellers, housewives or retired folks. Women, hence, tend to spend more time near their homes, giving them higher chances of observing sand mining boats and experiencing their impact first hand. In comparison, the male residents tend to travel out of the village to work, allowing them to network and discuss this topic with others. Similar findings were also reflected in Aaron^52^, where women expressed greater concern about climate change than men. Abedin^53^ also recognized that women in South Asia had limited mobility and ability to learn due to the gendered division of labour.

### Assessment of sand mining perception in the global context

People’s perceptions and awareness of sand mining in the VMD were similar to the findings of other global case studies. Overall, the most consistent finding was people’s awareness of the negative environmental impacts of riverbed sand mining, including land, air and water pollution, noise, land loss, and damage to roads and bridges. People can easily recognize the consequences of sand mining when it occurs in their vicinity. In terms of the perceived benefits of sand mining, although our study did not specifically seek details of income or economic contributions from sand mining jobs/industry, our results showed that most people agreed that sand mining provides jobs and the materials needed for local economic development. Consequently, most respondents expressed support for legal sand mining.

In our study, a number of respondents showed a lack of concern towards sand mining and related transportation activities, particularly if these activities did not directly disrupt their daily lives. While they were aware of the negative consequences of sand mining, these respondents felt powerless and had no means to raise their concerns with the local governments.

Finally, our results also show that those respondents who lived in areas that have directly suffered from riverbank erosion/collapse in the past exhibited a clear understanding of how sand mining contributed to bank erosion/collapse. These respondents also tend to be better educated and have learnt about the effects of sand mining from books and watching television. Engaging in discussions with others further enhanced people’s understanding of the impacts of sand mining.

## Conclusion

Our study interviewed 34 residents who lived along the Bassac River within An Giang Province and Can Tho City of the Vietnamese Mekong Delta (VMD). As we wanted to assess residents’ perception and awareness about sand mining and riverbank erosion, our respondents were specifically chosen based on their residency in areas affected by bank erosion or collapse. Our findings allow us to draw two main conclusions.

- Residents were supportive of riverbed sand mining, and their attention to sand mining activities and the mechanism driving riverbank erosion was primarily due to their own houses or lands being directly affected by bank erosion/collapse. These respondents who have been affected by sand mining were aware of the association of sand mining with bank erosion/collapse. They recognise the potential risk for future collapse and have actively sought to understand the underlying causes and mechanisms to effectively deal with bank erosion issues.
- Most residents were aware that river sand mining activities frequently happened along the length of the Mekong and Bassac Rivers. They understood that sand mining activities had caused various environmental issues such as pollution, noise, land loss, and damage to roads but at the same, the sand mining industry also provided jobs and income. Similar perceptions of sand mining were also recorded in other locations worldwide. However, only a few residents were able to link bank erosion/collapse to sand mining activities. Instead, many claimed that waves from passing boats were responsible for riverbank erosion. Subsequent land losses spurred some local residents to relocate their homes to more stable ground if they were adequately compensated by the local authorities. Those who were unable or refused to move will remain vulnerable to future bank erosion/collapse.

### Supplementary Information

Supplementary Information.

## Data Availability

Data could be acquired from the corresponding author on reasonable request.
