# Supplementary material for: Uncovering the lack of awareness of sand mining impacts on riverbank erosion among Mekong Delta residents: insights from a comprehensive survey
Source: Sci Rep. 2023 Sep 24;13:15937. doi: 10.1038/s41598-023-43114-w (PMC10518306; doi:10.1038/s41598-023-43114-w)
Supplement: Supplementary file 1 — Supplementary Information. [file 41598_2023_43114_MOESM1_ESM.docx]

**Uncovering the lack of awareness of sand mining impacts on riverbank erosion among Mekong Delta residents: Insights from a comprehensive survey**

Dung Duc Tran^1,2^, Nguyen Duc Thien^2^, Kai Wan Yuen^1^, Rachel Yu San Lau^1^, Jingyu Wang^1^, Edward Park^1*^

^1^ National Institute of Education (NIE), Earth Observatory of Singapore (EOS) and Asian School of the Environment (ASE), Nanyang Technological University (NTU), Singapore.

^2^ Centre of Water Management and Climate Change (WACC), Institute for Environment and Resources (IER), Vietnam National University, Ho Chi Minh City, Vietnam.

*Corresponding author: [edward.park@nie.edu.sg](mailto:edward.park@nie.edu.sg)

# **Supplementary Information**

**Table S1*.*** Sand mining perception across countries worldwide

| **ID** | **Publication Information** | **Findings** | **Compared with our studies** | **Group** |
| --- | --- | --- | --- | --- |
| 1 | Sustaining urbanization while undermining sustainability: the socio-environmental characterization of coastal sand mining in Lagos Nigeria (2022) in Lagos, Nigeria. The research method is interview, using ANOVA, PCA and quantitative data analysis framework^1^. | Small-scale sand mining took place over a long time. However, sand mining was a crucial economic source for developing infrastructure in the area. Sand mining negatively affected people's lives in terms of noise, dust, health, road damage, and erosion. It should be well organized and controlled. | People had a perception of negative impacts, but sand mining brought great economic benefits here then they have supported. Mining operations were problem-solving for long-term maintenance. | I  (more support, less protest) |
| 2 | Community Resistance against Sand Mining Activities at the Brown Canyon Semarang (2021) in Semarang, Indonesia. The research method is literature review, observation, and interview^2^. | Communities had less awareness and access to environmental issues (impacts, permits to exploit) but cares about the benefits made from mining (including sand mining). The political power and relations of the mine owner are great, and mining is not denied or resisted. Land used for farming is now barren due to exploitation. Environmental issues include soil pollution, water, gases, resource depletion, noise, road damage, and landslides. | People were less interested in environmental information. Besides, they were forced to favor more mining. Consequences include pollution of the environment and natural resources, noise, road damage, and landslides. | I  (more support, less protest) |
| 3 | Modelling the assessment of socio-economical and environmental impacts of sand mining on local communities: A case study of Villages Tatao River Bank in North-western part of Iran (2018) in Simineh, Iran. The research method is interview and applying LISREL model^3^. | The positive impact of sand mining (65.25%) was job creation and income, while 34.75% of people perceived negative effects (river bed destruction, degradation, conflicts, transport and traffic, agricultural land). The managers were less concerned about over-exploitation and illegality leading to environmental destruction and did not care about the people's protest. | People perceived economic benefits as more important than negative impacts. Managers were less interested in controlling riverbed sand mining and solving environmental problems. | I  (more support, less protest) |
| 4 | The Effects of Sand Mining on Rural Communities (2016) in Eastern Cape, South Africa. The research method is iinterview and focus group discussion^4^. | Most people perceived that sand mining brings jobs and income. Less people resisted when the government interfered with their livelihood and work while although they realized the conflicts, sea bank erosion, and health problems from mining activities. | People were highly aware of income and employment benefits from sand mining. People rebeled against the government when their income was affected. | I  (more support, less protest) |
| 5 | Livelihoods Built on Sand: Exposing the Precarity of Labour in Cambodia’s Sand Extraction Industry (2021) in Phnom Penh, Cambodia. The research method is experiment, observation and sustainability assessment^5^. | People had difficulty accessing environmental information (regulations and compensation). The law allowed uncontrolled exploitation and repression, making it difficult for dissidents. Mining was attractive because of jobs and incomes, and it was suitable for people away from home/migrants/people changing jobs due to reduced agricultural productivity. Environmental change due to sand mining affected agriculture. Erosion occurred due to sand mining and making migration. No insurance or regulation was proposed for workers. | People were aware of the negative environmental changes caused by sand mining but could not protest. It was because the power of the government allowed sand mining to take place smoothly, without control, and suppress the opposition. | II  (more protest, less support) |
| 6 | Paths of urban planning in a post-mining area. A case study of a former sandpit in southern Poland (2020) in Sosnowiec, Poland. The research method is interview and observation^6^. | The policy of planning development was most beneficial for land owners to exploit sand together and limited the decision-making capacity of the social community. The community was not consulted on future plans. The opinion of residents depended heavily on the assessment of the socio-economic situation. | Public opinion was limited, and managers favored decisions to develop the economic situation. | II  (more protest, less support) |
| 7 | Socio - Economic Impacts of Sand Mining Activities in Zanzibar (2020) in Zanzibar, Tanzania. The research method is literature review, observation, and group interview^7^. | Conflicts over agricultural land use for sand exploitation because only opinions were obtained from leaders, ignoring the people's views. The community was aware of both positive and negative impacts. The positive is income and work, and the negative is environmental issues on the ecosystem, reducing water resources, and agricultural land, increasing flood and soil erosion, community health, labor safety (mining accidents), and increased crime of illegal sand mining. | Most people negatively perceived sand mining but were powerless to protest and comment with mining companies. | II  (more protest, less support) |
| 8 | Effect of public perceptions on support/opposition of frac sand mining development (2019) in Trempeauleau, United State. The research method included email in-depth interview^8^. | Sand mining brings income but affects health (air and noise pollution), landscape, agricultural land, and increased traffic. Among the interviewers, 17% supported, 23% undecided but supported, 38% opposed, and 22% undecided but opposed. Oppose people supposed that sand mining does not bring high income and jobs because this is an area where people have a high rate of employment. | Most people perceived sand mining as having two sides, but most protest it because of its impact on agricultural land quality and health. Some people did not care about the problem; however, their perception favors environmental protection. | II  (more protest, less support) |
| 9 | The effect of community consultation on perceptions of a proposed mine: A case study from southeast Australia (2017) in Horsham, Australia. The research method is interview^9^. | Communities perceived their livelihoods and landscapes were being destroyed and felt powerless to stop or change the sand mining project. They were not treated equally by the mining company and were perceived as lacking in knowledge. The mining company did not provide complete information on environmental issues. Most community members have a negative perception of the consultation process and were not supportive of the mining project. | The community has a negative perception of mining (destroying livelihoods and landscapes) due to its inability to oppose the companies. They were treated unfairly. | II  (more protest, less support) |
| 10 | Perception based Study of Coastal Sand Dunes in the Areas of Ratnagiri Coasts, Maharashtra (2016) in Maharashtra, India. The research method is interview and observation^10^. | People were aware of the economic and environmental impacts of sand mining. The majority wanted to participate in managing and protecting the sand dunes. | People understood the two-side impacts of sand mining, and most wanted to protect the environment. | II  (more protest, less support) |
| 11 | Local perceptions on social-ecological dynamics in Latin America in three community-based natural resource management systems (2015) in Colombia, Mexico and Argentina. The research method is workshop with communities using Ostrom’s framework^11^. | Community-based natural resource management required external support and recognition to work effectively. The use of indigenous knowledge (Colombia), land use history (Mexico), and artisanal fishing history (Argentina) were all considered common challenges for community-based natural resource management. The government needed an effective environmental policy in the direction of resource exploitation. | Mining industry did not care about environmental policy in the management of resource exploitation. Community-based resource management was difficult. | II  (more protest, less support) |
| 12 | Report on state of Sand mining at Peri-Urban Kathmandu (2013) in Jhaukhel, Nepal. The research method applied observation and in-depth interview^12^. | Landslides caused by illegal sand mining and land cannot be used. After exploitation is completed, it cannot be recycled. There is lack of worker safety and environmental impacts, including landslides, reduced runoff, depletion of groundwater, and problems of drastically reduced flows and mudslides into farmland. Hence, 75% of local people opposed sand mining, the rest supported because of their jobs included. | Sand mining caused landslides/erosion due to illegal mining. Landowners/miners earned income but affect nearby residential areas' habitat, agricultural land, and water depletion. Many people opposed sand mining. | II  (more protest, less support) |
| 13 | Understanding the Power Interactions between Villages and the State: Insights from Sand Mining Issues (2020) in Beicun, China. The research method is observation^13^. | Villagers did not simply resist or obey state authority, but gradually sought a balance between the traditional ceremonial order and the modern political system. | The community seeked a balance in sand mining assessment decision-making with the authorities. | III  (balanced) |
| 14 | Public Perception toward the Impact of People Activities in Sand and Stone Mining on Economy and Environment in Nulokla Village Jayapura (2017) in Nolokla, Indonesia. The research method involved observations and interview^14^. | The industry generated worker income but has caused various environmental impacts, including reduced water flow, degraded soil, and erosion. Due to measurement, it is necessary to have policies and support to ensure workers' health. | Sand mining was an activity with both positive and negative sides. The negative side was effects on the soil and water environment and people's health. | III  (balanced) |
| 15 | Socio-economic assessment of riverbank erosion from heavy boat traffic: A case study at the Cho Gao Canal, Tien Giang, Vietnam (2022) in Tien Giang, Vietnam. The research method is interview, using WTA and LVI^15^. | People affected by boat traffic related to river bank erosion. | People were vulnerable due to the effects of river bank erosion. Restoration costs were estimated. | (IV)  Not available in perception |
| 16 | Assessing Social Vulnerability to Riverbank Erosion across the Vietnamese Mekong Delta (2022) in An Giang, Vietnam. The research method is interview^16^. | Migration was a direct result of landslides/river bank erosion. When landslides occurred, most people received assistance to evacuate and find work from neighbors and the government. The social vulnerability index (SVI) was used to assess demographic sensitivities, economic capacity, social networks, contact information, remitted income, access to finance, and the ability to receive support from/ to the neighbors. Demographic characteristics include age (elderly), dependent, low income, and low education. | This study evaluated the awareness of VMD people about river bank erosion. People are passively aware of responding to riverbank erosion. People received support (mainly financial) from neighbors and the government when moving. | (IV)  Not available in perception |
| 17 | Sand mining effects, causes and concerns: A case study from Bestari Jaya, Selangor, Peninsular Malaysia (2011) in Bestari Jaya, Malaysia. The research method is Experiment and hydraulic model of HEC-RAS^17^. | Environmental effects included turbidity, riparian habitats, flora and fauna, reduced sedimentation and increased "hungry" water, degraded roads, increased bank erosion, decreased water quality, and increased temperature. Measures were recommended to minimize the impacts before, during, and after exploitation. Illegal sand mining happened in many parts of Malaysia severely affected the river. | The study mainly evaluated the influence of sand mining on river morphology. | (IV)  Not available in perception |

**Table S2.** Questionnaire of the impacts of sand mining

| **Part A: Location** | | | | | | | | | | | | | | | | |
| --- | --- | --- | --- | --- | --- | --- | --- | --- | --- | --- | --- | --- | --- | --- | --- | --- |
| Province: | | District: | | | | | | | | Commune: | | | | | | |
| **Part B: General** | | | | | | | | | | | | | | | | |
| 1. | Full name | | | Gender □ Male □ Female | | | | | | | | | | | | |
| 2. | Age | | □ <30 | □ 30-60 | | | | | | | | □ >60 | | | | |
| 3. | Occupation | | □ Farmer | □ Fisherman | | | | | | | | □ Others: | | | | |
| **Part C: Role of river** | | | | | | | | | | | | | | | | |
| 4. | Does the river play an important role in your life? | | □ Yes | □ No | | | | | | | | | | | | |
| 5. | What do you use the river for? | | □ None  □ Fish, river food | □ Irrigation □ Transport | | | | | | | | □ Washing □ Others: | | | | |
| 6. | Did you see any change to the river recently? | | □ No change | | | | | | | | | | | | | |
|  |  |  | Fish | | | | | | □ More | | | □ Less | | | | |
|  |  |  | Water quality | | | | | | □ Clean | | | □ Dirty | | | | |
|  |  |  | Salinity, salt | | | | | | □ More | | | □ Less | | | | |
|  |  |  | Water level | | | | | | □ Higher | | | □ Lower | | | | |
|  |  |  | Riverbank collapse | | | | | | □ More | | | □ Less | | | | |
|  |  |  | Flooding | | | | | | □ More | | | □ Less | | | | |
|  |  |  | □ Others: | | | | | | | | | | | | | |
| **Part D: Sand mining** | | | | | | | | | | | | | | | | |
| 7. | Have you seen sand mining boats along the river? (show picture) | | □ Yes | | | | | | | □ No | | | | | | |
| 8. | How often do you see the boats? | | □ Never □ Everyday | | | | | | | □ A few times a month  □ Once every few months | | | | | | |
| 9. | Is there more sand mining recently or no change? | | □ More | □ Less | | | | | | | | | | □ No change | | |
| 10. | When did you start seeing sand mining boats? | | □ <5 years ago | | | □ 5-10 years ago | | | | | | | | □ >10 years ago | | |
| 11. | Which time do these sand mining boats mostly work per day? | | □ all day | □ morning | | | | | | □ afternoon | | | | □ (over) night | | |
| 12. | How many sand-carrying boats did you see per day? | | □ None | □ < 10 | | | | | | □ 10-30 | | | | □ > 10 | | |
| 13. | Have you seen trucks transporting sand along the roads? (show picture) | | □ Yes | | | | | | | □ No | | | | | | |
| 14. | How often do you see the trucks? | | □ Never □ Everyday | | | | | | | □ A few times a month   □ Once every few months | | | | | | |
| 15a. | Is sand mining good? | | □ Yes | | | | | | | □ No | | | | | | |
| 15b. | If yes, why? | | □ Money/ job | | □ Development | | | | | | | | □ Others: | | | |
| 16a. | *Do you think these effects are related to sand mining?* | | □ Pollution (land, water, air)  □ Less fish/shrimp…  □ Less vegetation  □ Water saltier | | | | | □ Noisier  □ Less animals  □ Damage to roads, bridges, buildings  □ Less flooding | | | | | | □ Bank erosion  □ Land loss  □ Disease  □ Less water from well | | |
| 16b. | *Have you experienced any of other impacts of sand mining?* | |  | | | | | | | | | | | | | |
| 17. | On a scale of 1 to 5, how bad are the problems?  1: Not so bad 5: Very bad | | Pollution (land, water, air) | | | | 1 | | | 2 | 3 | | | | 4 | 5 |
|  |  |  | Noisier | | | | 1 | | | 2 | 3 | | | | 4 | 5 |
|  |  |  | Bank erosion | | | | 1 | | | 2 | 3 | | | | 4 | 5 |
|  |  |  | Less fish/shrimp… | | | | 1 | | | 2 | 3 | | | | 4 | 5 |
|  |  |  | Less animals | | | | 1 | | | 2 | 3 | | | | 4 | 5 |
|  |  |  | Land loss | | | | 1 | | | 2 | 3 | | | | 4 | 5 |
|  |  |  | Less vegetation | | | | 1 | | | 2 | 3 | | | | 4 | 5 |
|  |  |  | Damage to roads, bridges, buildings | | | | 1 | | | 2 | 3 | | | | 4 | 5 |
|  |  |  | Disease | | | | 1 | | | 2 | 3 | | | | 4 | 5 |
|  |  |  | Water saltier | | | | 1 | | | 2 | 3 | | | | 4 | 5 |
|  |  |  | Less flooding | | | | 1 | | | 2 | 3 | | | | 4 | 5 |
|  |  |  | Less water from well | | | | 1 | | | 2 | 3 | | | | 4 | 5 |
|  |  |  | Others: | | | | 1 | | | 2 | 3 | | | | 4 | 5 |
| 18. | Did you try to solve the problems from sand mining? | | □ Yes | | | | | | | □ No | | | | | | |
| 18a. | If yes, what did you do? | |  | | | | | | | | | | | | | |
| 18b. | If no, why did you not do anything? | |  | | | | | | | | | | | | | |
| 19. | Do you think sand mining is illegal? | | □ Yes | | | | | | | □ No | | | | | | |
| 20. | Any other comments? | |  | | | | | | | | | | | | | |
| 21 | Please kindly provide us your phone number | |  | | | | | | | | | | | | | |

# **Supplementary Reference**

1. Aliu, I. R., Akoteyon, I. S. & Soladoye, O. Sustaining urbanization while undermining sustainability: the socio-environmental characterization of coastal sand mining in Lagos Nigeria. *GeoJournal* **87**, 5265–5285 (2022).

2. Wicaksana, A. S. & Amirudin, A. Community Resistance against Sand Mining Activities at the Brown Canyon Semarang. *E3S Web Conf.* **317**, 01037 (2021).

3. Farahani, H. & Bayazidi, S. Modeling the assessment of socio-economical and environmental impacts of sand mining on local communities: A case study of Villages Tatao River Bank in North-western part of Iran. *Resour. Policy* **55**, 87–95 (2018).

4. Mngeni, A., Musampa, C. M. & Nakin, M. D. V. The effects of sand mining on rural communities. *Sustain. Dev. Plan. VIII* **1**, 443–453 (2016).

5. Arragon, L. van. Livelihoods Built On Sand: Exposing the Precarity of Labour in Cambodia’s Sand Extraction Industry. (University of Ottawa, 2021).

6. Krzysztofik, R., Dulias, R., Kantor-Pietraga, I., Spórna, T. & Dragan, W. Paths of urban planning in a post-mining area. A case study of a former sandpit in southern Poland. *Land use policy* **99**, 104801 (2020).

7. Ali, A. S. Socio - Economic Impacts of Sand Mining Activities in Zanzibar. (The Open University of Tanzania, 2020).

8. Hammond, E. A. S. Effect of public perceptions on support/opposition of frac sand mining development. *Extr. Ind. Soc.* **6**, 471–479 (2019).

9. Walsh, B., van der Plank, S. & Behrens, P. The effect of community consultation on perceptions of a proposed mine: A case study from southeast Australia. *Resour. Policy* **51**, 163–171 (2017).

10. Sapkale, J. B. & Rathod, B. L. Perception based study of coastal sand dunes in the areas of Ratnagiri coasts, Maharashtra. *Indian J. Sci. Technol.* **9**, 1–7 (2016).

11. Delgado-Serrano, M. del M. *et al.* Local perceptions on social-ecological dynamics in latin america in three community-based natural resource management systems. *Ecol. Soc.* **20**, (2015).

12. Sada, R. & Shrestha, A. Report on State of Sand Mining at Peri-Urban Kathmandu: Case of Jhaukhel VDC. (Nepal Engineering College, 2013).

13. Huang, Q. & Xu, J. Understanding the Power Interactions between Villages and the State: Insights from Sand Mining Issues. *Asian J. Soc. Sci.* **48**, 567–587 (2020).

14. Ratang, S. Public Perception toward the Impact of People Activities in Sand and Stone Mining on Economy and Environment in Nulokla Village Jayapura. *J. Educ. Vocat. Res.* **8**, 45–48 (2017).

15. Anh, H. H. & Thuy, N. Socio-economic assessment of riverbank erosion from heavy boat traffic: A case study at the Cho Gao Canal, Tien Giang, Vietnam. *IOP Conf. Ser. Earth Environ. Sci.* **967**, 12005 (2022).

16. Tri, V. P. D., Trung, P. K., Trong, T. M., Parsons, D. R. & Darby, S. E. Assessing social vulnerability to riverbank erosion across the Vietnamese Mekong Delta. *Int. J. River Basin Manag.* 1–31 (2022).

17. Ashraf, M., Maah, M., Yusoff, I., Wajid, A. & Mahmood, K. Sand Mining Effects, Causes and Concerns: A Case Study from Bestari Jaya, Selangor, Peninsular Malaysia. *Sci. Res. essays* **6**, 1216–1231 (2011).
